# The Role of Probiotics in Cancer Treatment: Emphasis on their *In Vivo* and *In Vitro* Anti-metastatic Effects

**DOI:** 10.22088/acadpub.BUMS.6.2.1

**Published:** 2017-05-31

**Authors:** Elahe Motevaseli, Ali Dianatpour, Soudeh Ghafouri-Fard

**Affiliations:** 1 *Department of Molecular Medicine, School of Advanced Technologies in Medicine, Tehran University of Medical Sciences, Tehran, Iran.*; 2 *Food Microbiology Research Center, Tehran University of Medical Sciences, Tehran, Iran.*; 3 *Department of Medical Genetics, School of Medicine, Shahid Beheshti University of Medical Sciences, Tehran, Iran.*

**Keywords:** Probiotics, lactobacilli, cancer, metastasis, invasion

## Abstract

Probiotics are defined as live bacteria and yeasts that exert beneficial effects for health. Among their various effects, anti-cancer properties have been highlighted in recent years. Such effects include suppression of the growth of microbiota implicated in the production of mutagens and carcinogens, alteration in carcinogen metabolism and protection of DNA from oxidative damage as well as regulation of immune system. We performed a computerized search of the MEDLINE/PUBMED databases with key words: cancer, probiotics, lactobacilli, metastasis and invasion. Cell line studies as well as animal models and human studies have shown the therapeutic effects of probiotics in reduction of invasion and metastasis in cancer cells. These results support the beneficial effects of probiotics both *in vitro* and *in vivo*. However, pre-clinical or clinical studies are not enough to decide about their application.

Lactic acid bacteria including the genus *Lactobacillus* and *Bifidobacterium* have been shown to exert beneficial effects in human (1). Numerous lines of evidence have shown that changed gut microbiota is associated with several common disorders including cancer. Therefore, resuming the equilibrium using the beneficial bacteria (called “probiotics”) for disease treatment and prevention has been regarded profitable (1). Probiotic bacteria have recently become the focus of research because of their anti-cancer properties. The underlying mechanisms for their anti-cancer effects are versatile including suppression of the growth of microbiota implicated in the production of mutagens and carcinogens, alteration in carcinogen metabolism, and protection of DNA from oxide damage as well as regulation of immune system (2). In addition, they have been shown to change expression of different genes participating in cell death and apoptosis (3), invasion and metastasis (4), cancer stem cell maintenance (5) as well as cell cycle control (6). Further studies have shown their modulatory effects on the cancer-related signaling pathways in a cell type specific manner (7-9). In addition, their anti- proliferative effects have been assessed in several cell line studies (10-12). Notably, a traditional fermented milk product has been shown to inhibit* in vitro *proliferation of MCF-7 breast cancer cells but not normal mammary epithelial cells which implies that the bioactive substances prompt responses that are specifically detected in tumor cells (13). Special attention has been given to the effects of probiotics in reduction of invasion and metastasis in cancer cells in cell line studies as well as animal models and human studies. Invasion and metastasis have been regarded as important hallmarks of malignant cells which are endowed to them through diverse and complex genetic or epigenetic aberrations as well as extrinsic signals, such as those relayed from their microenvironment (14). The schematic description of various molecules and cells involved in metastasis are summarized in Figure 1. Metastasis cascade include acquisition of the ability to interrupt the basement membrane, invasion into the stroma (local invasion), passing the blood circulation (intravasation), staying alive in the circulation before they can reach to a remote organ, and production of clinically evident metastases. During this process, cancer cells recruit numerous stromal cells to support them in each step. Consequently, cancer microenvironment not only participates in the early steps of carcinogenesis but also contributes in metastasis cascade (15). Several studies have assessed the effects of probiotics on critical steps of invasion and metastasis such as interruption of cell–cell adhesion, epithelial-mesenchymal transition, tumor microenvironment, and cancer stem cell maintenance. The results of these studies have been summarized in the following sections.

**Evidence acquisition**

We performed a computerized search of the MEDLINE/PUBMED, Web of Knowledge, Scopus, ProQuest and Google Scholar databases with key words: cancer, probiotics, lactobacilli, metastasis, and invasion within the maximal date range until 2017.

**Cell–cell adhesion**

Tight junction between epithelial and endothelial cells has a critical role in preserving cell to cell integrity. Defects in this structure underlie the invasion and thus metastasis process (16). Tight junction structure has several molecular components including zona occludens-1 (ZO-1), claudin-1, and occludin. Effective assembly and preservation of this structure is carried out through the anchorage of the transmembrane proteins by the peripheral or plaque proteins such as ZO-1. Indeed, this protein provides a scaffold to fix a number of tight junction molecules together (16). On the other hand, the matrix metalloproteinases (MMPs) are regarded as critical participants of cell invasion through their role in degradation of various extracellular matrix proteins which enables cancer cells to migrate and invade (17). Considerably, cell-free supernatants (CFS) from *L. casei* and *L. rhamnosus* GG have been shown to prevent colon cancer cell invasion suggesting that probiotic CFS has anti-metastatic bioactive substances that may participate in cell in vation decrease *in vitro* (18). Such decrease in cell invasion has been later found to be accompanied by a decrease in matrix metalloproteinase-9 (MMP-9) protein level in cultured metastatic human colorectal carcinoma cells and an increase in the level of the tight junction protein ZO-1 in cultured metastatic human colorectal carcinoma cells (19). In addition, perioperative probiotic treatment has been shown to maintain the liver barrier in patients undergoing colorectal liver metastases surgery (20). A more recent study has shown that *L. rhamnosus* and *L. crispatus* CFSc can decrease expression of matrix metalloproteinase-2 (MMP-2), MMP-9 in HeLa cells and increase expression of their inhibitors. *L. rhamnosus* showed this effect in HT-29 cells as well (4). Furthermore, *L. acidophilus* and *L. rhamnosus* GG have been shown to regulate MMP-9 expression by the up-regulation of tissue inhibitor of metalloproteinases (TIMP)-1 and down-regulation of CD147 in phorbol 12-myristate 13-acetate- differentiated human monocytes (21). CD147 is over-expressed in numerous tumor cells and enhances metastasis formation by induction of both angiogenesis and MMPs expression (22, 23). On the other hand, TIMP-1 is tissue inhibitor of MMPs and its up-regulation has resulted in the inhibition of MMP-2 and suppression of metastasis (24). Recently, it has also been reported that *L. rhamnosus* GG significantly down-regulates expression *GLUT1* in the MDA-MB-231 cells (8). This gene encodes an important rate-limiting protein in the transport of glucose into cancer cells. Its inhibition has been shown to decrease MMP-2 expression and c-Jun NH2-terminal kinase (JNK) activation, which controls numerous targets in the metastatic cascade (25). Lipoteichoic acid (LTA) deficient *L. acidophilus* (NCK2025) has been shown to increase *ICAM5*, *RUNX3*, *TIMP2*, *RASSF1A* expression in human colon carcinoma cell line HT-29 (26). *ICAM5* codes for a type I transmembrane glycoprotein that is a member of the intercellular adhesion molecule family. It has been shown to be highly methylated in a fraction of colon cancer specimens. Its methylation diminishes the cell-to-cell adhesion in the cancer cells leading to enhancement of invasive potential (27). RUNX3 inhibits cancer cell migration and invasion through up-regulation of TIMP-2, which successively prevents MMP-2 expression and function (28). RASSF1A is a genuine tumor suppressor protein that can enhance death receptor-dependent cell death through TNF-R1, TRAIL or Fas activation (29). Moreover, its methylation has been shown to be associated with colorectal cancer development (30). Considering the role of *L. acidophilus* (NCK2025) in restoration of expression of mentioned tumor suppressor genes, this probiotic might be efficient in suppression of metastasis. Another study has demonstrated that probiotic conditioned media treatment diminished the up-regulation of genes in the NF-κB activation pathway, and down-regulated genes participating in extracellular matrix remodeling including MMPs, tissue-type plasminogen activator urokinase (PLAU) and its receptor (PLAUR) (31). Additionally, Kefir as a probiotic-containing fermented milk product has been shown to exert cytotoxic effects on 4T1 breast tumor cells. A notable decrease in tumor size and weight, a considerable enhancement in helper T cells and cytotoxic T cells as well as significant decreases in metastasis to lung and bone marrow were detected in the kefir water-treated BALB/c mice after 4T1 cancer cells transplantation (32). Kefir has been shown to exert an anti‑proliferative effect on Caco‑2 and HT‑29 cells, and is accompanied by induction of cell cycle arrest at the G1 phase, induction of apoptosis, up-regulation in Bax:Bcl‑2 ratio and an increase in p53 independent‑p21 expression, while it does not influence either the motility and invasion of these cells *in vitro* or MMP expression (33). In an *in vitro* model of the human epithelium, *L. plantarum* prompted translocation of ZO-1 to the tight junction region. Besides, *L. plantarum* has been demonstrated to initiate Toll-like receptor 2 (TLR2) signaling, and treatment of Caco-2 monolayers with the TLR2 agonist enhanced translocation of occludin in the tight junction (34). Recently, *L. rhamnosus* GG has been shown to improve intestinal integrity by inhibition of miR122a leading to occludin restoration in Caco-2 colorectal cancer cells (35). Furthermore, viable *L. rhamnosus* GG could significantly up-regulate ZO-1, Claudin-1 and Occludin gene expression in Caco-2 cells leading to restoration of destroyed epithelial barrier (36). *L. reuteri* I5007 has been shown to exert similar effects in the expression of tight junction related proteins in newborn piglets (37).  Another study has assessed the ability of Caco-2 cells to degrade collagen matrix and passing from membrane following treatment with different concentrations of probiotic bacteria. Notably, *L. acidophilus *and *L. casei* supernatants and cell extracts have decreased cell invasion capacity. Invasion inhibition effect of *L. acidophilus *was more than that of *L. casei *(38). As targeting tumor cell motility within the primary tumor is capable of prevention local invasion (39), colonization of *lactobacilli* in the site of the primary tumor may be beneficial in the prevention of metastases.

**Fig 1:**
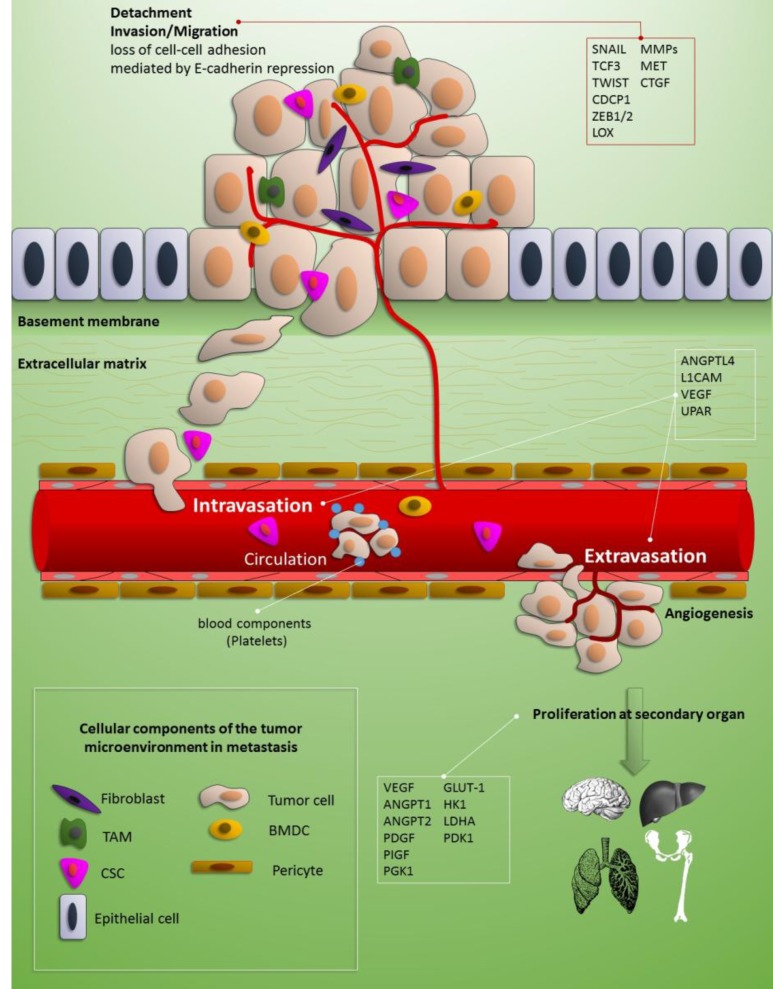
The schematic description of various molecules and cells involved in the metastasis. Metastasis cascade include loss of cell-cell adhesion, acquisition of the ability to interrupt the basement membrane, invasion into the stroma (local invasion), passing the blood circulation (intravasation), staying alive in the circulation before they can reach to a remote organ, extravasation and production of clinically evident metastases. Several molecules are involved in each step. TAM: tissue activated macrophage; CSC: cancer stem cell; BMDC: bone marrow derived cells

**Epithelial-mesenchymal transition (EMT)**

EMT is a biological process that permits a polarized epithelial cell, which typically interacts with basement membrane through its basal surface, to undertake numerous biochemical alterations which result in acquisition of a mesenchymal cell phenotype. Such phenotype change is accompanied by increased migratory capacity and invasiveness (40). Among the different factors and pathways involved in EMT, stromal cell-derived factor 1 (SDF-1) and its receptor, CXCR4 have gained special attention. CXCR4 has been shown to enhance EMT through the Wnt/β-catenin signaling pathway. Thus, targeting of the SDF-1/CXCR4 axis has been suggested as a treatment strategy in cancer suppression (41). Anti-CXCR4 antibodies have been shown to inhibit CXCL12 mediated cancer cell adhesion, migration, and proliferation (42). Notably,* L. acidophilus* NCFM has been shown to exert anti-metastatic effects via down-regulation of CXCR4 expression in colon, mesenteric lymph nodes and spleen of tumor-bearing mice (43). Considering the role of MMPs in the maintenance of EMT (17), the observed role of *lactobacilli* in down-regulation of MMPs (4) implies a putative role for them in suppression of EMT.

Live *L*. *casei* has been demonstrated to induce apoptotic cell death in both murine (CT26) and human (HT29) colon carcinoma cell lines as well as an experimental tumor model. Tumor growth inhibition has been associated with up-regulation of the TNF-related apoptosis-inducing ligand TRAIL (10).  Previously, it has been determined that soluble *TRAIL *gene and actinomycin D synergistically inhibit metastasis of TRAIL-resistant colon cancer in the liver (44). Also, trail resistance has been shown to trigger EMT and increase breast cancer cell invasiveness by modulation of PTEN and miR-221 expression (45). However, there is some contradictory evidence regarding the role of TRAIL in metastasis in other cancers such as pancreatic ductal adenocarcinoma. In this cancer, TRAIL prompted the expression of the proinflammatory cytokines as well as urokinase-type plasminogen activator and increased the invasion cancer cells *in vitro* (46).

*L. casei* and *L. rhamnosus* GG have been

demonstrated to suppress NF-κB activation by the inhibition of IκBα destruction in intestinal epithelial cells (47-49). Besides, bacteria-free solution originating from *L. plantarum* has been shown to suppress various NF-κB pathways (50). As NF-κB activity is associated with EMT and metastatic potential in various cancers (51, 52), the modulation of this pathway by certain lactobacilli strains may be of practical value.

**Tumor microenvironment**

Tumor microenvironment is constructed via the interactions between tumoral and non-transformed cells. The latter have an active and often tumor-promoting role at all stages of tumorigenesis. The major non-malignant cell types that are detected in this microenvironment are the cells of the immune system, the tumor vasculature and lymphatics, as well as the fibroblasts, pericytes and adipocytes (53). Many animal studies have shown that the beneficial anti-metastatic effects of lactobacilli are accompanied by or exerted via modulation of microenvironment. For instance, *L. casei* YIT9018 has been shown to suppress pulmonary and regional lymph node metastases in mice and guinea pigs (54). Intralesional injection of *L. casei* YIT9018 in highly metastatic melanoma bearing C57BL/6 mice has been shown to suppress tumor growth and improve the survival of affected animals. In addition, intravenous (I.V.) injection of this strain protects the mice against pulmonary metastasis after I.V. injection of melanoma cells. Injection of these lactobacilli exerts protective effects against both the axillary lymph node metastasis and lung metastases depending on the route and timing of injections. These effects are accompanied by augmentation of natural killer (NK) cell activity as well as cytolytic activity of axillary lymph node cells (55). Another study has shown that lymph node cells activated by the subcutaneous injection of these lactobacilli participate in the suppression of the metastasis (6). Matsuzaki et al. have reported that intralesional injection of *L. casei* YIT9018 into Lewis lung carcinoma-bearing mice suppresses both the growth of the primary tumors and the development of lung metastases. In the *L. casei* YIT9018-primed mice, intraperitoneal administration of *L. casei* elicits a high level of IL-2 and IFN-γ in the peritoneal cavity and enhances host immune response against tumor (56). Yazdi et al. have shown that selenium nanoparticle-enriched *Lactobacillus brevis* (*L. brevis*) elicits efficient immune responses in tumor bearing BALB/c mice, decreases the liver metastasis in metastatic form of mouse breast cancer and improves the life span of animals' life span. The immune responses include an increase in the level of IFN-γ and IL-17 as T helper 1 cytokines and enhancement in the activity of NK cells (57). Aragon et al. have demonstrated that the administration of milk fermented by *L. casei* CRL 431 diminishes or inhibits tumor growth with less tumor vascularity, extravasation of tumor cells, and lung metastasis. These benefits are accompanied by alterations in the immune response such as decreasing the infiltration of macrophages in both the tumor and the lungs and an increased antitumor response associated to CD8+ and CD4+ lymphocytes (58). Takagi et al. have detected anti-metastatic effects of *L. casei* Shirota (LcS) in transplantable tumor cells which is mediated through augmentation of NK cells cytotoxicity (41). *L. rhamnosus* GG has been shown to exert effective antioxidative activity *via* diminishing reactive oxygen species production and phagocytic capacity of the neutrophils (59). Considering the role of neutrophils in almost all steps of cancer metastasis which is exerted in response to tumor-derived incitements (60), the inhibition of their function by probiotics might be an efficient strategy which impedes metastasis. Furthermore, a constituent of polysaccharide peptidoglycan complex on *LcS* has been shown to exert beneficiary effects in murine model of inflammatory bowel disease and colitis-associated cancer through inhibition of IL-6/STAT3 signaling (61). Considering the constitutive activation of STAT3 in many cancers and its fundamental roles in different steps of metastasis cascade such as cell transformation and migration, angiogenesis, as well as modulation of tumor microenvironment (62), its down-regulation by lactobacilli might affect metastasis potential of cancer cells. Likewise, a recent study has shown that kefir water exerts antiangiogenic effects in breast cancer through down-regulation of the IL-1β angiogenic factor that promotes tumor invasiveness, as well as the vascular endothelial growth factor (VEGF) which is a crucial mediator for angiogenesis (32). Further, decrease of the proangiogenic factor IL-6 has been detected following treatment with probiotics in breast cancer models (58, 63-65). All data presented above support the role of probiotics in changing pro-tumoral microenvironment.

**Cancer stem cells**

The presence of a fraction of multipotent “cancer stem cells (CSC)” in solid tumors as well as hematological malignancies has resulted in suggestion of a new model for explanation of tumorigenesis process (42). These cells are thought to directly or indirectly participate in the induction of metastasis. Furthermore, the heterogeneity detected in CSCs has resulted to suggest a role for them in determination of complexity and organ specificity in metastases (66). Many transcription factors as well as signaling pathways are implicated in the maintenance of CSCs. Among them are the hypoxia inducible factors (HIFs) which facilitate transcriptional responses to regional hypoxia in normal tissues and in cancers. Also, they induce specific signaling pathways and transcription factors, such as Notch and Oct4, which are implicated in stem cell self-renewal and multipotency (43). Notably, *L. rhamnosus *has been shown to down-regulate the expression of HIF-1α in MDA-MB-231 triple negative breast cancer cell lines (8). Considering the specific activation of HIF-1α signaling in the stem cells of mouse lymphoma and human acute myeloid leukemia and the effect of their inhibitors in preferential eradication of CSCs in mouse models (38), modulation of HIF-1α signaling following treatment with lactobacilli might be of therapeutic value. Another study has revealed that a combination of eight Gram-positive bacterial strains (*Streptococcus thermophilus*, *Bifidobac-terium longum*, *Bifidobacterium breve*, *Bifidoba-cterium infantis*, *L. acidophilus*, *L. plantarum*, *L. casei*, and *L. bulgaricus*) could activate NK cells to provide enhanced differentiation of CSCs which finally has led to suppression of tumor growth, and decreased inflammatory cytokine release (40). In addition, we recently detected the over-expression of *SFRP2*, an antagonist of Wnt pathway in HT-29 colorectal cancer cells following *L. rhamnosus* treatment and in HeLa cells following *L. rhamnosus* and *L. crispatus* treatments. Takig into the account the involvement of Wnt-induced CSCs in colorectal cancer metastasis (67) as well as the role of SFRP1 in the inhibition of the transformation and invasion abilities of cervical cancer cells via modulation of Wnt signal pathway (68), lactobacilli can be considered as putative therapeutic modalities in these cancer types.

## Discussion

Even with extensive work committed to the early diagnosis and prevention of cancer, micro- or macro- metastases exist in most diagnosed patients at the time of their referral to diagnostic settings. In particular, metastasis is regarded as a possible life span-dependent destiny for both the early and late stage cancer patients (55). Consequently, several studies have focused on finding substances with anti-metastatic properties. For this purpose, it is necessary to find tumor and host factors contributing in the metastasis cascade. The “seed and soil” hypothesis suggested by Paget in 1889 (69) is now extensively assented in the scientific literature (70). The progenitor cell, initiating cell, cancer stem cell, or metastatic cell are now considered as the “seed”, whereas host factors, stroma, or the organ microenvironment are regarded as the “soil” (70). The consequence of metastasis is reliant on the communication between tumor cells and receptive tissues (70). Probiotics have been shown to influence all cell types and pathways implicated in the metastasis. Previously, lactobacilli-based immunotherapy has been suggested to be used along with conventional therapeutics to overcome the failures of the traditional treatment options, especially in the treatment of cancer metastases (2). As discussed formerly, the beneficial effects of lactobacilli in the cancer therapy are not confined to their immunomodulatory effects. They have been shown to alter expression of several genes involved in cell transformation, migration and invasion. Besides, it should be emphasized that the antimetastatic properties of probiotics might be different in distinct species of these organisms. Future studies are needed to identify putative pathways or molecules that are target of strain-specific gene expression modulation. Additionally, identification of formulations with the best bioactivity and less side effects is another challenge in this regard. Probiotic lactobacilli have also been shown to protect against cyclophosphamide-caused myelo-suppression in animal models which has led to the improvement of the resistance to *Candida albicans.* Consequently, probiotics have been suggested as a modality to decrease immunosu-ppression in cancer patients (71). Moreover, a randomized control study in critically diseased children has shown that the intake of probiotics decreases the occurrence of acute infectious, nosocomial and antibiotic-associated diarrhea in numerous general pediatric situations (72). Nevertheless, in some immuno-compromised patients, there have been occasional cases of sepsis following probiotics intake (73). In brief, the results of recent studies on evaluation of the effects of probiotics on cancer cell invasion and metastasis have supported their beneficial effects both *in vitro* and *in vivo*. Nonetheless, pre-clinical or clinical studies are not enough to decide about their application.

Consequently, to translate the results of basic studies to clinical application and to avoid unwanted side effects, the exact component of lactobacilli which is responsible for beneficial effects should be determined in pre-clinical animal studies. Although the possibility of synergic effects of different components should be considered as well, some studies have revealed contradictory effects for different lactobacillus-derived substances (19). Additional studies for the identification of the bioactive components and their mechanism of action could lead to the application of probiotics as a nutritional modality to prevent metastasis.

## Conflict of interest

Authors declare no conflict of interest.
